# High-resolution bathymetries and shorelines for the Great Lakes of the White Nile basin

**DOI:** 10.1038/s41597-022-01742-3

**Published:** 2022-10-22

**Authors:** Stuart E. Hamilton, David D. McGehee, Chrispine Nyamweya, Collins Ongore, Amina Makori, Richard Mangeni-Sande, Esther Kagoya, Benedicto B. Kashindye, Mboni Elison, Sophia S. Shaban, Enock Mlaponi, Venny M. Mwainge, Henry Ocaya, Noah Krach, Zachary Ogari, Bairon Mugeni, Anthony Taabu-Munyaho, Robert Kayanda, Elias Muhumuza, Vianny Natugonza

**Affiliations:** 1 Department of Coastal Studies, East Carolina University, Greenville, NC 27858 USA; 2 Emerald Ocean Engineering LLC, 107 Ariola Drive, Pensacola Beach, FL 32561 USA; 3 Kenya Marine and Fisheries Research Institute (KMFRI), Kisumu Center, Kisumu, Kenya; 4 National Fisheries Resources Research Institute (NaFIRRI), PO Box 343, Jinja, Uganda; 5 Tanzania Fisheries Research Institute (TAFIRI), PO Box 475, Mwanza, Tanzania; 6 Department of Geography and Geoscience, Salisbury University, Salisbury, USA; 7 Fisheries Office, Kenya Fisheries Service, Nairobi, Kenya; 8 Lake Victoria Fisheries Organization (LVFO), PO Box 1625, Jinja, Uganda; 9 Busitema University Maritime Institute, Namasagali, Uganda

**Keywords:** Hydrology, Environmental sciences, Geography, Water resources

## Abstract

HRBS-GLWNB 2020 presents the first open-source and high-resolution bathymetry, shoreline, and water level data for Lakes Victoria, Albert, Edward, and George in East Africa. For each Lake, these data have three primary products collected for this project. The bathymetric datasets were created from approximately 18 million acoustic soundings. Over 8,200 km of shorelines are delineated across the three lakes from high-resolution satellite systems and uncrewed aerial vehicles. Finally, these data are tied together by creating lake surface elevation models collected from GPS and altimeter measures. The data repository includes additional derived products, including surface areas, water volumes, shoreline lengths, lake elevation levels, and geodetic information. These data can be used to make allocation decisions regarding the freshwater resources within Africa, manage food resources on which many tens of millions of people rely, and help preserve the region’s endemic biodiversity. Finally, as these data are tied to globally consistent geodetic models, they can be used in future global and regional climate change models.

## Abstract (summary)

**Table:** 

Measurement(s)	bathymetry • shorelines
Technology Type(s)	echo soundings • remote sensing
Sample Characteristic - Environment	lake bed • lake shore
Sample Characteristic - Location	Africa • East Africa • Uganda • • Tanzania • Democratic Republic of the Congo • Lake Victoria • Lake Edward • Lake Albert • Lake George

## Background

**Fig. 1:**
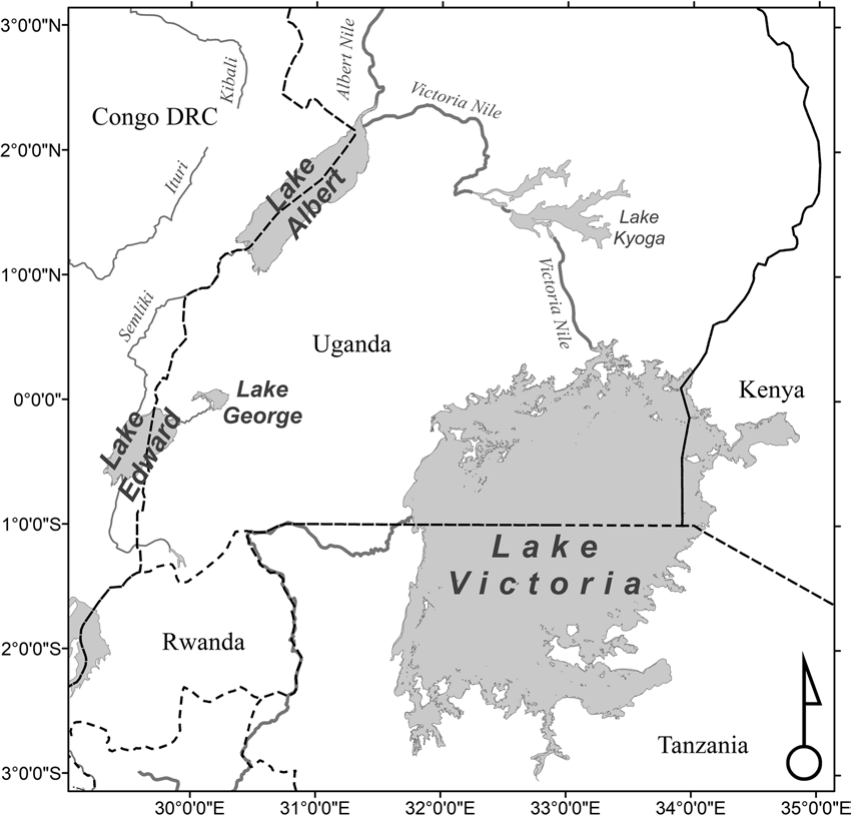
Study Area. The combined study areas across all Lakes.

Lakes Victoria, Albert, Edward, and George are situated across Uganda, Tanzania, Kenya, and the Democratic Republic of Congo (DRC) (Fig. 1). All the Lakes aside from Victoria are in an active continental fault and rift zone within the East African Rift System^1^. Lakes Albert, Edward, and George are located within a half-graben, distinguished by normal rift faults. Lake Victoria, however, sits in a localized depression in a relatively low-lying area between the raised rift shoulders of the Eastern and Western branches of the East African Rift System ^1^.

These lakes constitute the primary freshwater inputs of the White Nile and contain much of the freshwater holdings of East Africa. Attempts to better manage freshwater resources across arid regions of east and northeast Africa; attempts to manage regionally important fisheries across East Africa; attempts to preserve the endemic native freshwater species of East Africa; as well as attempts to build improved regional climate models are all hindered by inadequate detailed information on the water resources and characteristics of these Lakes. The current geospatial data lack the spatial and temporal fidelity to be incorporated into global and regional models and assist decision-making. Lakes Albert, Edward, and George lack coherent lake-wide data, whereas Lake Victoria has limited data, but much of it is from almost a century ago.

From a hydrologic perspective, the Nilean Great Lakes discharge an average of 47 km^2^ of freshwater annually into the White Nile^2^ at the northern tip of Lake Albert (Fig. 1). The freshwater inputs from the Nilean Great Lakes provide consistent water inputs into the Nile system as opposed to the larger but seasonal freshwater inputs from the Blue Nile. The freshwater flowing from these Lakes provides much of the required water to sustain the year-round agriculture in northern Uganda, South Sudan, Sudan, and Egypt. Despite numerous international agreements, the Nile River system remains a source of potential international water conflicts across numerous nations^3^. The data in this repository add important information about these critical lakes and their water capacities at the Niles’ source.

Each of the four Lakes has a thriving local fishery on which millions of residents rely. As of 2019, the annual catches of Lake Albert and Lake Edward are estimated to be 31,384.8 tons and 32,092.8 tons, respectively^4^, and almost all the shoreline residents rely on this overstretched fishery for their livelihood^5^. Lake George has a significant local fishery with eight landings sites across the Lake and the attached Kazinga Channel. Across Lake Victoria, an *Oreochromis niloticus* aquaculture industry is exploding^6^ alongside the well-established *Oreochromis niloticus*, *Lates nilotics*, and *Rastrineobola argentea* wild fisheries. In 2014, the total production across all fisheries in Lake Victoria was estimated at USD 650 million^7^, even in a period of declining stocks^8^. Indeed, Lake Victoria is likely the single most crucial freshwater fishery in Africa^9^. The data in this repository add much-needed information to help manage these critical fisheries.

From a biodiversity perspective, it is estimated that 78.2 percent of the freshwater fishes are endemic within the Lake Victoria basin. This endemic percentage is likely higher if the undescribed endemic haplochromine cichlids are included^10^. Unfortunately, these endemic haplochromines have suffered catastrophic declines over the last 70 years^11–13^, causing a mass extinction within the Lake. By 1991, it was estimated that two-thirds of endemic haplochromines in Lake Victoria were either extinct or threatened with extinction^14^, out of an estimated five hundred or more endemic species once present^13^. However, the deep-water regions of the Lake, where the remaining endangered haplochromine species likely reside, are poorly delineated and lack granular bathymetric data of the type provided in this repository.

During 2017 and 2020, we undertook a project to map the shorelines, bathymetry, sediment, and other associated data for Lakes Victoria, Albert, Edward, and Lake George (Fig. 1). In addition, hydroacoustic surveys, shoreline delineations, water-level measures, and geodetic surveys were conducted across all four lakes. The motivation behind this data collection effort was to provide information to help preserve the native biodiversity of the Lakes and support sustainable fisheries.

## Methods

The survey of Lake Albert was conducted in February 2020. The Lake Edward and Lake George surveys were conducted in August of 2020. The surveys of Lake Victoria were conducted between September and November of 2017, 2018, 2019, and 2020. We assume no significant morphological change occurred in Lake Victoria across these 4-years. All collection periods correspond to the end of a traditional dry season and the transition period into the beginning of a traditional wet season. Water levels were monitored during the period of each Lakes’ survey. Benchmarks were installed during each Lakes survey aside from Lake Victoria, where an existing benchmark nail existed. Unmanned aerial systems (UAS) were flown during the Lake Albert survey to assess our shoreline delineation methodology.

### Lake elevation levels

Lake Victoria utilizes spaceborne altimetry to ascertain its lake elevation. Lakes Albert, Edward, and George have no systematic high accuracy spaceborne altimeter measure of lake elevations. Therefore, Lake Albert, Edward, and George’s lake elevations are derived from statistical analyses of observed water levels.

#### Lake sounding datums

For Lake Albert, Lake Edward, and Lake George, visual water-level (WL) observations taken throughout the survey are averaged to obtain the lake elevation (LE), also known as the project sounding datum (SDp). The method for determining SDp is to observe the WL on a graduated board, often called a tide board or a staff gauge (G), securely attached to a piling or other solid vertical structure extending below the lake surface. The graduations are then marked relative to the gauge zero (G_0_). The WL is read as the distance above or below G_0_ where the water surface intersects the gauge.

A fixed, tamper-resistant benchmark (Bm) was installed or in operation at each Lake within the optical leveling distance of each gauge to achieve the conversion from local water levels to ellipsoidal heights and EGM 2008 elevations. First, each Bm’s horizontal and vertical position was measured using the

Global Navigation Satellite System (GNSS). Then, the vertical distance between the benchmark elevation (BmE) and G_0_ is measured using standard optical or laser-based survey methods. This distance is the vertical gauge offset (VGO).

**Fig. 2:**
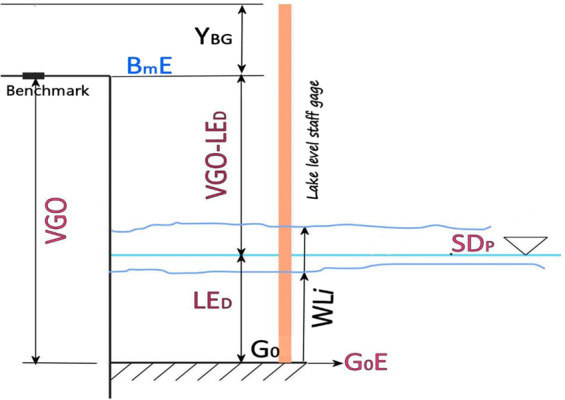
Lake Elevation (SDp). This diagram represents the relationship between the various Lake Elevation parameters directly measured (in black text), obtained from instruments (in blue text), or calculated (red text).

The Lakes’ elevation methodology is summarized in Fig. 2 and is defined in Eq. 1. At this point, SDp for Lakes Albert, Edward, and George is merely an ellipsoidal height; the ellipsoidal height is converted to EGM:2008 using Harmonic Synthesis at the horizontal coordinate location of each Bm^15,16^.

Eq. 1 - Lake Albert, Lake Edward, and Lake George Sounding Datums

$${\rm{SDp}}=\left({\rm{Bm}}+{\rm{VGO}}+WL\right)$$

SDp is the lake elevation or project sounding datum, Bm is the benchmark elevation from RTK GPS, VGO is the vertical gauge offset derived by using an optical level, and WL is the water level obtained from the gauge reading.

Unlike Lake Albert, Lake Edward, and Lake George, due to Lake Victoria’s size, with a maximum diameter exceeding 375 km, hydrodynamic effects could readily negate the hydrostatic assumption that the lake surface is uniformly level. On Lake Victoria, wind setup, seiching, and the significant outflow into the Victoria Nile would result in hydraulic gradients that would make any single, nearshore water level gauge unrepresentative of lake levels at points distant from the gauge. To establish a meaningful SDp for Lake Victoria using nearshore water level gauges, at least three stations distributed equilaterally around the Lake’s perimeter would need to be established and operated simultaneously for long periods. However, this approach was deemed unfeasible primarily due to cost and logistical constraints. For example, creating a multi-country concurrent network of gauges would require at least three times the equipment, three times the labor, and three times the training.

The alternate approach utilizes Jason-3 spaceborne altimeter data. This method has been used in Lake Victoria and is supported by the USDA G-REALM program^17^. Jason-3 is a radar altimeter launched in January 2017. The primary goal of Jason-3 is to provide sea-level variations with accuracies under 2.5 cm at a repetition cycle of 10-days^18^. As Jason-3 passes over Lake Victoria, it can establish EGM 2008 elevations for the Lake from numerous measures towards the middle of the Lake. Jason-3 passes over 150 km of Lake Victoria. The collection path runs from approximately Nyabansari in Tanzania to Bugaia in Uganda. As the instrument is radar-based, climatic conditions rarely limit the data collection. The raw altimeter data collected by Jason-3 undergoes numerous corrections before a lake surface elevation is determined, including a dry tropospheric correction, a wet tropospheric correction, an ionosphere correction, and an instrument-specific bias adjustment^19^. Lake elevation observations were obtained from Jason-3 during Lake Victoria’s surveys across 2017, 2018, 2019, and 2020. The average of the Jason-3 readings from 2020, which itself is an average of many hundreds of observations, defines the *SDp* for the Lake Victoria surveys.

A Lake Victoria benchmark is still surveyed at a water level gauge to allow for past and future data integration, and the benchmarks are tied to the altimeter measures used. At this point, SDp for Lake Victoria is already in EGM:2008 as Jason-3 uses EGM:2008 as opposed to ellipsoidal elevations, so harmonic synthesis is not required as it is for the other Lakes.

##### Lake benchmarks

Benchmarks for Lake Albert (BmA), Lake Edward (BmEd), and Lake George (BmG) were installed along each of the three Lakes’ shorelines. Each benchmark is situated within a few meters and line-of-sight of a water level staff gauge. A preexisting benchmark nail (BmV) located above the gauge was utilized for Lake Victoria. Aside from Lake Victoria, each installed benchmark is an 8 cm diameter brass disc stamped with LEAF II. Each installed benchmark was anchored approximately 15 cm into a larger concrete pad using a twisted steel reinforcement bar. Each benchmark’s location was obtained using long-term GNSS averaging, captured by a Hemisphere GNSS receiver with Atlas satellite-based augmentation system wide-area corrections applied. Observations without a corrective signal were discarded. Ellipsoidal elevation, recorded to the millimeter level, was also captured by the GPS receiver. Conversion of benchmark ellipsoid elevations to EGM 2008 WGS 1984 Version used the harmonic synthesis coefficients provided by the U.S. National Geospatial-Intelligence Agency (NGA) EGM Development Team^15,16^.

*BmA* was installed on 1/31/2020 within the UPDF Marine compound at Mbegu, approximately 6.5 km east-northeast of Kaiso, Uganda, on the eastern side of Lake Albert. Across seven days between 2/1/2020 and 2/20/2020, the horizontal location of the benchmark was recorded by a GNSS receiver with built-in averaging. The GPS unit averaged horizontal locations at the benchmark until it reached 95 percent confidence. In addition, the ellipsoidal height was collected on the surface of Lake Albert across the survey period and adjusted to the benchmark elevation using the vertical gauge offset and the water level readings. The total number of vertical observations is 35,550.

*BmEd* was installed on 2/13/2020 at the fish landing site in Katwe Village, Uganda, at the northern end of Lake Edward. Across portions of 8/5/2020, 8/10/2020, 8/13/2020, and 8/15/2020, one X, Y, and Z GPS location were recorded every 5-seconds, totaling 11,242 observations.

*BmG* was installed on 8/11/2020 at the landing site in Kahendero, Uganda, on the western side of Lake George. On 8/13/2020, one X, Y, and Z GPS location were recorded every 5-seconds, totaling 2,663 observations. Unfortunately, *BmG does* not have a full unobstructed 360° view of the sky and may require further refinement.

A preexisting benchmark nail (BmV) at the railroad dock in Jinja, Uganda, is used for Lake Victoria. The nail is located directly above the water level gauge and marked with a white paint X. Across portions of 3/22/2021 and 3/23/2021, one X, Y, and Z GPS location was recorded every 5-seconds, totaling 6,842 observations. Still, as noted earlier, altimetry data was used for the actual SDp.

##### Lake gauges

Within a few meters of each benchmark, a water level staff gauge was either installed or already existed. For Lake Victoria (GV), Lake Albert (GA), and Lake Edward (GE), preexisting gauges were used. At Lake George (GG), a temporary gauge was established for the duration of field operations.

*GA* is a staff gauge of unknown origin. The staff is a simple iron square tube painted decimeter intervals subdivided into 5 cm steps. The 100 cm subdivision at the top of the gauge was surveyed relative to the BmA (Fig. 2, Y_BG_) using an optical level on 1/31/2020. Between 2/1/2020 and 2/20/2020, twelve lake level observations were collected. The water level only varied by 6 cm across the entire survey. The average of the 12-daily readings was used to help define the SDp for the Lake Albert bathymetric survey.

*GE* is a long-term gauge installed by the Ugandan Ministry of Water. The gauge is a stepped gauge consisting of three separate concrete pillars of increasing height with graduated measurement strips attached at the centimeter level. The water level on the gauge, relative to the BmEd, was surveyed using an optical level on 8/10/2020. Twice-daily Lake level observations continued throughout the 11-day survey operation between 8/5/2020 to 8/22/2020. The water level only varied by 3 cm across the entire survey. The average of the 11-daily readings was used to help define the SDp for the Lake Edward bathymetric survey.

*GG* is a temporary gauge installed for the duration of field operations. The gauge is a simple wooden gauge with painted centimeter intervals anchored to a galvanized steel pipe driven between 1 m and 2 m into the substrate. The water level on the gauge, relative to the BmG, was surveyed using an optical level on 8/12/2020. Once-daily Lake level observations were collected across the two days of the hydrographic survey and the day before and after the survey. The water was stable across the entire survey. The two average daily readings were used to define the SDp for the Lake George bathymetric survey.

*GV* is a long-term gauge installed by the Ugandan Ministry of Water. The gauge has graduated measurement markers at the two-centimeter level. The zero level on the gauge, relative to the BmV, was surveyed on 3/22/2021 and 3/23/2021. As B*m*V and GV are at the same horizontal coordinates, leveling is not required. Water level observations were not utilized from this gauge during the survey, as the Jason-3 altimeter was used to establish the Lake elevation level for Lake Victoria. Instead, the closest four Jason-3 measures across the survey dates are used to calculate the water level. The water level varied by 4 cm across the 2017 bathymetric survey, 9 cm across the 2018 survey, 5 cm across the 2019 survey, and 13 cm across the 2020 survey. The 2020 water level is used as the SDp to allow for as close as possible temporal consistency across all Lakes in the database.

##### Lake elevation data

Table 1 provides each lake’s SDp in the most common gravitational models and all input parameters to the lake elevation models. The SDp for Lake Edward is 915.77 m (EGM08), the E/SDp for Lake George is 915.74 m (EGM08), and the SDp for Lake Albert is 622.18 m (EGM08), and the SDp for Lake Victoria is 1136.92 m (EGM08). Measures of uncertainty are provided in the technical validation.

**Table 1:** Lake Level Parameters for each Lake.

	BENCHMARK (m)					GAUGE (m)		LAKE ELEVATION (m)		Model
	Easting	Northing	Ellipsoid	EGM Adj	BME	VGO	G_0_	WL	LE/SDp	
E	820898.09	9983581.78	905.28	−11.36	916.64	2.67	913.97	1.80	915.77	EGM 2008^15^
	820898.09	9983581.78	905.28	−9.96	915.24	2.67	912.57	1.80	914.38	EGM 96^45^
	820898.09	9983581.78	905.28	−8.96	914.24	2.67	911.56	1.80	913.37	EGM 84^45^
A	279969.50	171117.28	606.67	−15.52	622.70	0.8	620.30	0.28	622.18	EGM 2008^15^
	279969.50	171117.28	606.67	−13.80	620.99	0.8	618.59	0.28	620.47	EGM 96^45^
	279969.50	171117.28	606.67	−9.93	617.11	0.8	614.71	0.28	616.60	EGM 84^45^
G	171927.68	5538.53	906.14	−10.74	916.87	2.02	914.85	0.89	915.74	EGM 2008^15^
	171927.68	5538.53	906.14	−9.81	915.95	2.02	913.93	0.89	914.82	EGM 96^45^
	171927.68	5538.53	906.14	−9.21	915.35	2.02	913.33	0.89	914.22	EGM 84^45^
V	850925.68	41035.54	1123.75	−13.9	1137.70	1.71	1135.99	0.93	1136.92	EGM 2008^15^
	850925.68	41035.54	1123.75	−13.85	1137.60	1.71	1135.89	0.93	1136.82	EGM 96^45^
	850925.68	41035.54	1123.75	−11.76	1133.80	1.71	1134.73	0.93	1134.73	EGM 84^45^

### Lake bathymetries

The Lake Albert hydroacoustic survey was conducted across 14-days between February 1^st^, 2020, and February 20^th^, 2020. The Lake Edward hydroacoustic survey was conducted across 10-days between August 4^th^, 2020 and August 22^nd^, 2020. On August 13^th^, 2020 and August 14^th^, 2020, the Lake George hydroacoustic survey occurred during a Lake Edward Survey break. The Lake Victoria hydroacoustic survey occurred daily between September 8^th^, 2017 and October 7^th^, 2017, September 10^th^, 2018 and October 9^th^, 2018, September 15^th^, 2019 and October 13^th^, 2019, and finally between October 20^th^, 2020 and November 25^th^, 2020. The Lake Victoria soundings from 2017, 2018, and 2019 were vertically corrected to align to the 2020 water levels. The earlier year were adjusted by 1.28 m (0.03 m, 95CI), 0.975 m (0.06, 95CI), and 1.025 m (0.05 m, 95 CI), respectively.

The hydroacoustic survey transect designs were based on local topography, available bathymetry, and cost considerations. Both Lake Albert and Lake Edward had dominant relief patterns running from the Congolese highlands in the west to the Ugandan Plateau in the east, forming a deep U shape perpendicular to the Albertine Rift. The survey transects were designed to follow this axis of high relief across the Albertine Rift. Lake George and Lake Victoria have no discernable relief patterns, both being relatively shallow bowls situated across flat planes. Therefore, the survey designs were optimized to capture an adequate portion of these two Lakes while minimizing cost.

#### Lake soundings

Across Lake Albert, Lake Edward, and Lake George, a 9 m, V-bottomed, shallow draft research vessel was deployed with a Ugandan crew out of Jinja, Uganda. The echosounder used to collect the soundings was a dual-frequency sounder with a built-in data logger, external GNSS receiver, and a combined low-frequency (33 kHz) high-frequency (200 kHz) transducer. Both frequencies were operational and recorded during the survey, but only the high-frequency signal was processed to produce Lake Albert and Lake George’s soundings. Greater than 90 percent of Lake Edward also used the high-frequency sounder, but the instrument was switched to low-frequency in areas over 90 m deep. A speed of sound adjustment was made based on the water sampling that occurred on average twice each transect. Calibration was performed before the initial deployment.

For Lake Albert, Lake Edward, and Lake George, Hydromagic 9.1 software was used to record and process the acoustic soundings into tabular X, Y, and Z formats. The echosounder’s echogram was output in real-time to a laptop. A dedicated 12-volt battery, maintained by a 60-watt solar panel mounted on the cabin top, powered all equipment. Positions were obtained by a multi-frequency GNSS antenna connected to the echosounder. The transducer was mounted on an aluminum extension pole that supported the GNSS antenna directly above the transducer. The antenna received Atlas L-band satellite-based augmentation system (SBAS) correction signals that allow precise positioning.

Lake Victoria soundings were collected by the stern trawler RV Lake Victoria Explorer by members of the Hydroacoustics Regional Working Group of the Lake Victoria Fisheries Organization. This group is based out of Jinja, Uganda, Kisumu in Kenya, and Mwanza in Tanzania. This group has conducted twenty-three acoustic surveys of Lake Victoria since 1999 under an established protocol^20^. The RV Explorer is a 17 m research vessel and a V-shaped hull with a draft of 1.8 m. The echosounder used on the RV Explorer is a dual-frequency system operating at 70 kHz and 120 kHz, respectively. The transducers are mounted on a protruding instrument keel under the boat and powered by the vessel’s electrical system. Calibration was performed immediately before each daily survey. The GPS logger used on this system is not differentially corrected.

For Lake Victoria, Echoview 8.0 software was used to record and process the soundings into tabular X, Y, and Z formats. After noise was removed from the raw signal and adjustments were made to correct the beam angle, the initial lakebed soundings were obtained using the best bottom candidate algorithm^21^. A CTD probe was used at each calibration site to determine the local environmental conditions. The average water temperature at the calibration site was input into the system to predict the sound speed. Lake Victoria’s survey’s calibration protocol is detailed in the Standard Operating Procedures for Hydroacoustics surveys on Lake Victoria^20^.

**Fig. 3:**
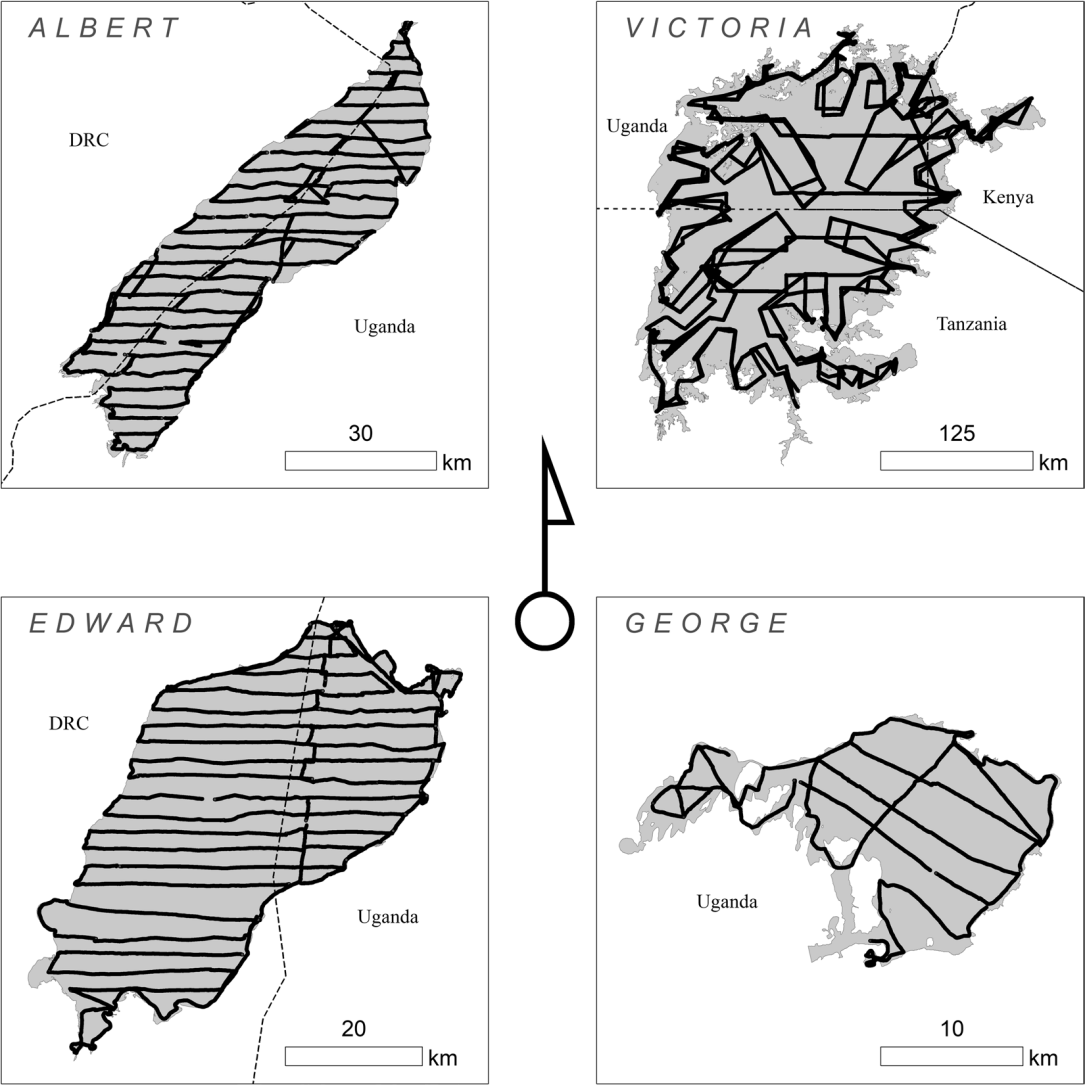
Project Soundings. All soundings across all Lakes.

Across all Lakes, either a certified coastal engineer or an individual with relevant expertise processed the echograms from the echosounder. The process essentially involves detecting the average bottom in the echogram and digitizing through small peaks and pits caused by the boat’s motion. A narrow interpretation is needed on calm days, and the automated extraction of the lake bottoms often suffices. On days with rough water, manual digitization of the trace is required. Sometimes, the signal may reflect off anything in its path to the bottom, including suspended sediment, debris, animals, subaquatic vegetation, silt, mud, or a harder compacted layer beneath a softer surface layer. The digitization process removes such anomalies as well as smoothing over dropouts and other noise. Finally, the digitized trace is exported to tabular soundings for use in GIS and other software. Figure 3 represents the soundings across all Lakes.

#### Lake bathymetries data

For Lake Albert, Lake Edward, and Lake George, the output spatial and tabular data contains; the date of the sounding, the horizontal position of the sounding, and corrected depth using a local-verified speed of sound adjustment for both high-frequency and low-frequency soundings when applicable, the vessel speed at the time of the sounding, the vessel heading at the time of the sounding, and a field indicating if the GNSS was operating in uncorrected or corrected mode for each sounding. For Lake Victoria, the output spatial and tabular data contains the date of the sounding, the time of the sounding, the horizontal position of the sounding, corrected depth using a local-verified speed of sound adjustment, and a field indicating if the GNSS was operating in uncorrected or corrected mode for each sounding. Depth zero corresponds to the LE /SDp for each Lake as already defined.

Across Lake Albert, 290,018 soundings were collected (Table 2), resulting in 53 soundings per square kilometer. Across Lake Edward, 225,528 soundings were collected (Table 2), resulting in 101 soundings per square kilometer. Across Lake George, 59,281 soundings were collected (Table 2), resulting in a density of 211 soundings per square kilometer. Finally, across Lake Victoria, 17,958,859 soundings were collected (Table 2), resulting in a density of 269 soundings per square kilometer. The water volume and mean depth are calculated using constrained Delaney Triangulation, whereas the maximum depth is the deepest collect sounding. The summary information for each Lakes’ bathymetry is shown in Table 2 and is compared against values from the (WLD) World Lakes Database^22^ unless otherwise noted.

**Table 2:** Bathymetry Characteristics.

	Average Depth (m)		Max Depth (m)		Volume (km3)		Soundings Count
	GLWNB	WLD^22^	GLWNB	AGLI^46^	GLWNB	WLD^22^	GLWNB
E	32.10	17	114.40	112	80.39	39.52	225,528
A	35.82	25^46^	57.88	25	174.23	280^46^	290,018
G	2.13	2.4	6.25	4^47^	0.60	0.8	59,281
V	39.77	40	74.12	80	2,302.19	2760^48^	17,958,859

### Lake shorelines

For each of the Lakes, we constructed high-resolution shorelines from spaceborne imagery at a combination of 15 m, 10 m, 5 m, 3 m, 50 cm, and 30 cm. Accuracy statistics were generated using UAS-derived imagery at 10 cm.

#### Sentinel-2 imagery

Sentinel-2 is designed to map and monitor water cover, inland waterways, and coastal areas^24^. The baseline spaceborne imagery used to delineate the shorelines across Lake Albert, Lake Edward, and Lake George is Sentinel-2. Sentinel-2 is a European Space Agency (ESA) wide-swath, high-resolution (HR), a multi-spectral imaging system that consists of two satellites flying in the same orbit but phased at 180°^23^. The system carries an optical instrument payload that samples thirteen spectral bands: four bands at 10 m resolution, six bands at 20 m resolution, and three bands at 60 m resolution^25^. The four bands at 10 m resolution are centered on the wavelengths 0.490 µm, 0.56 µm, 0.665 µm, and 0.842 µm, respectively. These wavelengths correspond to the blue, green, red, and near-infrared portions of the electromagnetic spectrum. These spectral properties of Sentinel-2 allow for color composites and false color composites of each of the Lakes at 10 m resolution. Furthermore, as the radiometric signal in the near-infrared band is almost entirely absorbed by open water, it can assist in delineating a water-terrestrial edge boundary.

The Sentinel-2 data granules used to delineate the Lake Albert shoreline are:

- S2B_MSIL1C_20190403T080609_N0207_R078_T36NUH_20190403T110906, S2B_MSIL1C_20190503T080619_N0207_R078_T36NTG_20190503T112849, S2B_MSIL1C_20190503T080619_N0207_R078_T36NTH_20190503T112849, S2B_MSIL1C_20190503T080619_N0207_R078_T36NUG_20190503T112849
  The Sentinel-2 data granules used to delineate the Lake Edward shoreline are:
- MSIL1C_20170702T081009_N0205_R078_T35MRV_20170702T082404, MSIL1C_20170821T080959_N0205_R078_T35MQV_20170821T082855
  The Sentinel-2 data granule used to delineate the Lake George shoreline is:
- S2B_MSIL1C_20191229T081239_N0208_R078_T35NRA_20191229T100818

#### Landsat imagery

The baseline spaceborne imagery used to delineate the Lake Victoria shoreline is Landsat-8. Landsat-8 is a USGS/NASA, high-resolution (HR), multi-spectral imaging system. Landsat-8 uses a push-broom Operational Land Imager and Thermal Infrared Sensor to collect data with a spatial resolution of 30 meters in the visible and near-infrared regions of the electromagnetic spectrum. The relevant bands at 30 m resolution are the blue band located between 0.45 µm to 0.51 µm, the green band located between 0.53 µm to 0.58 µm, the red band located between 0.64 µm to 0.67 µm, and the near-infrared band located between 0.85 µm to 0.88 µm. As the infrared band is almost entirely absorbed by open water, it can assist in delineating a water-terrestrial edge boundary. In addition, a 15 m panchromatic band is located between 0.64 µm to 0.67 µm and is used to pansharpen the 30 m bands to allow for feature digitizing at 15 m resolution. These spectral properties of Landsat-8 allow for color composites and color-infrared composites of Lake Victoria at 15 m resolution when pan-sharpened.

The Landsat data are listed below.

LC81700602020049LGN00, LC81700602021003LGN00, LC81700612020001LGN00, LC81700612020049LGN00, LC81700622020017LGN00, LC81700622020049LGN00. LC81710602020040LGN00, LC81710602021026LGN00, LC81710612020040LGN00, LC81710622020040LGN00, LC81720602020047LGN00

#### Very high-resolution planetscope eye imagery

In highly dynamic vegetative areas where Sentinel-2 or Landsat-8 cannot delineate a clear shoreline, very high resolution (VHR) imagery was obtained and used (Table 3). For example, the southern wetland of Lake Albert across both the DRC and Uganda uses 50 cm Worldview 2 (WV2) and 30 cm Worldview 3 (WV3) imagery as opposed to Sentinel-2 (Table 3), as this region has ephemeral floating grasses, sub-aquatic vegetation, and therefore shows a reflected signal response in the near-infrared bands of the satellite imagery. Thus, the wetland areas of Lake Albert are of substantially higher resolution than the rest of the Lake Albert shoreline.

**Table 3:** Shoreline Remote Sensing Instrument.

Instrument	Resolution (m)	Victoria	Albert	Edward	George
**Landsat 8**	30/15	x			
**Sentinel-2**	10	x	x	x	x
**PlanetScope**	3		x		
**WV2 50** **cm**	0.5		x		
**WV3 30** **cm**	0.3		x		
**UAS**	0.1		x		

#### Sub-meter resolution UAS

Finally, sub-meter resolution (SMR) UAS was flown over Lake Albert to ascertain the shorelines’ positional accuracies. Once the accuracy statistics were calculated, the UAS data was incorporated back into the shorelines for these areas. These UAS-derived shorelines are the regions around Kaiso, Butiaba, and Ntoroko on Lake Albert in Uganda.

#### Shoreline digitization

The initial step of the shoreline delineation was selecting the required satellite scenes—the selected scenes needed to meet the following criteria, be mostly cloud-free over the Lakes, and have suitable flags indicating high-quality data. The ESA Copernicus Hub and USGS GLOVIS sites were searched until the images met the above criteria. The selected granules were then subset only the Blue, Green, Red, and near-infrared bands, and the Landsat-8 imagery was pan-sharpened. Once composited, each 4-band raster is represented as a color-IR composite and a visible color composite. Before digitizing began, the resolution was set to 1:20,000 for all Lakes aside from Lake Victoria, which was set to 1:30,000.

Fishnets were constructed that covered the entirety of each Lake. The shoreline in each cell of the fishnet is manually digitized in a heads-up manner. The first pass of each cell digitizes the exterior shoreline of the Lake. The second pass of each cell digitizes all islands in the cell, and the third pass digitizes potential nearshore obstructions. Once each cell is complete, a second cartographer verifies the digitization and sends all questions back to the original digitizer, making the required updates. The final stage is to combine all the individual shoreline cells of the fishnet into a singular whole for each Lake and then verify the constructed shoreline feature’s topology.

#### Resolution and scale

Using Tobler’s rule of scale and resolution^26^, it is possible to create a shoreline that approximates 1:20,000 scale from the 10 m Sentinel-2 images and 1:30,000 from the Landsat-8 imagery using appropriate error monitoring and control. The Planet Scope imagery at 3 m resolution would equate to 1:6,000, the WV2 imagery at 50 cm resolution would equate to 1:1000, the WV3 imagery at 30 cm resolution would equate to 1:600, the UAS imagery at 10 cm resolution would equate to 1:200. For these reasons, the Lakes Albert, Edward, and George shorelines can be considered at a minimum 10 m resolution or a 1:20,000 scale product. The Lake Victoria shoreline can be regarded as a minimum 15 m resolution or a 1:30,000 scale product. We report the coarsest resolution as the shoreline’s resolution from the coarsest instrument, but large portions of the shorelines are higher resolution from less coarse instruments.

#### Lake shorelines data

We find the surface area of Lake Edward, Lake Albert, Lake George, and Lake Victoria to be 2,241,119,039 m^2^, 5,423,949,967 m^2^, 281,121,696 m^2^, and 66,792,882,259 m^2^, respectively. We find the shoreline lengths of Lake Edward, Lake Albert, Lake George, and Lake Victoria to be 241,395 m, 484,454 m, 89,204 m, and 3,063,755 m, respectively. The summary information for each Lakes’ shoreline is shown in Table 4, and the data are compared to the Global Self-Consistent, Hierarchical, High-Resolution Geography Database (GSHHG)^27^, considered the current best available consistent across these Lakes^27^.

**Table 4:** Shoreline Characteristics.

	Surface Area (m^2^)		Shoreline Length (m)		Islands		Vertices	
	GLWNB	GSHHG^27^	GLWNB	GSHHG^27^	GLWNB	GSHHG^27^	GLWNB	GSHHG^27^
**E**	2,241,119,039	2,212,536,133	329,789	241,395	42	0	9,216	406
**A**	5,423,950,008	5,421,142,762	589,726	484,454	79	0	59,564	833
**G**	281,121,697	298,236,653	218,328	89,204	17	3	4,517	153
**V**	66,792,882,229	69,057,499,142	7,142,068	3,063,755	985	78	119,025	5,122

### Hardware and Software

Soundings were collected and processed using Eye4Software Hydromagic or Echoview Software Pty Ltd, Echoview software. The sounding collection system used for Lake Albert, Lake Edward, and Lake George was the CEESystems CEESCOPE. High-frequency soundings for Lake Albert, Lake Edward, and Lake George were collected using a 200 Khz transducer from CEE Systems. The low-frequency soundings for the deep-water portion of Lake Edward were collected using a 33 kHz transducer from CEE Systems. The sounding collection system used on Lake Victoria before 2020 was a Simrad EK 60 dual frequency echo sounder with a 7° beam angle connected to 70 kHz and 120 kHz general-purpose dual transducer produced by Kongsberg Maritime AS. For 2020, the sounding collection system was changed to a Simrad EK80 dual frequency echo sounder, which operated at the same frequencies. The GNSS system used on Lake Albert, Lake Edward, and Lake George was a Novatel Hemisphere GPS. The Hemisphere Atlas system provided the SBAS L-Band GPS real-time correction. The Hemisphere Atlas system provided the SBAS L-Band GPS real-time correction. GNSS system used on Lake Victoria was a Globalsat Technology Corporation GPS.

ESRI ArcGIS ArcPro^28^, GDAL/OGR^29^, and QGIS^30^ were used to perform all horizontal coordinate transfers, conduct geostatistical analysis, produce cartographic outputs, digitize shorelines, post-process the soundings, and analyze the soundings. Microsoft Excel was used to process and transform the SDp GPS data. Harmonic synthesis transformation for data conversion to EGM 2008 was conducted in the *Harmonic Synth WGS 84* Fortran code provided by the NGA^15^.

Sentinel-2 and PlanetScope were the primary data sources for the satellite imagery The SenseFly EBee + UAS^31^, with the SODA survey camera^32^, was used to fly the data and then assess the accuracy of the shoreline delineation. SenseFly Emotion^33^ software was used to plan and fly all UAV missions. Pix4D^34^ was used to process all UAV imagery.

Tinfour 2.7.1^35^ to triangulate mass bathymetric soundings and calculate each Lakes’ mean depths and volume.

## Data Records

HRBS-GLWNB 2020 is publicly available as a geospatial repository in a Harvard Dataverse at https://dataverse.harvard.edu/dataverse/GLWNB-2020. The vector data are available in open shapefile format and the raster data in open GeoTiff format. Tabular data is available in CSV format. Due to the large file size, some of the datasets are compressed. The data are organized by Lake and data type.

All soundings for all Lakes are available in vector point and tabular format^36^. Attributes include the actual sounding, the horizontal location of the soundings, the GPS status of the soundings, and other ancillary data such as the date and time of the sounding. Bathymetric maps, derived from the soundings, are also available at this location^36^.

Each Lakes elevation (SDp), benchmark, and water-gauge information are in point and tabular formats^37^. All data are provided in ellipsoidal heights and EGM 84, EGM 96, and EGM 08. The data are organized by Lake and data type. Attributes include the actual lake level zero for each Lake, the horizontal location of the benchmark used to derive the lake level zero, each GPS point that feeds into the benchmark location, and the gravitational offsets used to convert each Lakes elevation from ellipsoidal height to an Earth Gravitational Model. Each Lakes shoreline is available as a vector polygon^38^. The repository includes geospatial metadata for each dataset.

## Technical Validation

The estimation of uncertainty takes a dual approach. Firstly, defining and attempting to measure each component’s uncertainty and then combining these uncertainty measures into a single statistical measure of uncertainty applicable across the entire dataset or, when possible, creating a higher-truth sample of the data and using this higher-truth sample to assess the population’s uncertainty using cross-validation.

### Lake elevation uncertainty (LEU)

The LEU uncertainty is the combined uncertainty of the benchmarks’ vertical locations, the water level readings, the error introduced when transferring data between the two, and the transfer of ellipsoidal heights to EGM 2008.

The 99 percent confidence interval for each of the benchmarks’ ellipsoidal height is expressed below and used as the ellipsoidal uncertainty (EU).

$$\begin{array}{lll}E{\rm{U}}\;G & = & 906.14\;m\pm 0.0121\;m\;(CI\;99,n=2,663)\\ E{\rm{U}}\;E & = & 905.28\;m\pm 0.0048\;m\;(CI\;99,n=11,242)\\ E{\rm{U}}\;V & = & 1123.75\;m\pm 0.0081\;m\;(CI{\rm{}}99,n=6841)\\ E{\rm{U}}\;A & = & 606.67\;m\pm 0.0035\;m\;(CI{\rm{}}99,n=35,\,550)\end{array}$$

Optical level uncertainty (OLU), in vertical millimeters, is the square route of the distance measured in kilometers multiplied by sixty^39^. The combined distance from the Lake Albert benchmark to the optical level and the Lake Albert gauge to the optical level is estimated at 20 m introducing a potential maximum leveling error of 0.008 m. The combined distance from the Lake Edward benchmark to the optical level and the Lake Edward gauge to the optical level is estimated at 20 m introducing a potential maximum leveling error of 0.008 m. The combined distance from the Lake George benchmark to the optical level and the Lake George gauge to the optical level is estimated at 40 m introducing a potential maximum leveling error of 0.012. The Lake Victoria optical measure error is 0 m, as the gauge location is at the same horizontal location as the benchmark, and the altimeter is used to determine the SDp.

Any single WL’s uncertainty (WLU) is assumed to be the scale uncertainty of one-half of each gauge’s gradation, which is 0.025 m for Lake Albert and 0.005 m for Lake Edward, Lake George, and Lake Victoria.

Jason-3 is a relatively new altimeter, and its application to inland water bodies is even more recent, but terrestrial water bodies evaluations are in development. Over Lake Issyk-Kul, the absolute bias of Jason-3 is reported as −28 mm ± 40 mm StD^40^. Across numerous French rivers, it has an RMSE between 0.20 m and 0.30 m^41^, and the instrument is designed to operate at 2.5 cm of accuracy^42^, and this value is used as the total LEU for Lake Victoria.

#### Lake levels combined uncertainty

For Lake Albert, Lake Edward, and Lake George, the LEU uncertainty is calculated using the Root Sum of the Squares GUM methodology^43^ (Eq. 2). For Lake Victoria, the LEU is merely the uncertainty of the Jason-3 Lake elevation as both the benchmark and gauge are bypassed (Eq. 2).

Eq. 2 - SDP uncertainty

$$\begin{array}{rll}LEU & = & \sqrt{E{U}^{2}+OL{U}^{2}+WL{U}^{2}}\\ LEU\;A & = & \sqrt{0.003{5}^{2}+0.00{8}^{2}+0.02{5}^{2}}\\  & = & 0.03\;m\end{array}$$

$$\begin{array}{lll}LEU\;E & = & \sqrt{0.004{8}^{2}+0.00{8}^{2}+0.0{5}^{2}}\\  & = & 0.01\;m\end{array}$$

$$\begin{array}{lll}LEU\;G & = & \sqrt{0.012{1}^{2}+0.01{2}^{2}+0.00{5}^{2}}\\  & = & 0.02\;m\\ LEU\;V & = & 0.03\;m\end{array}$$

where LEU = Total SDp Uncertainty, V = Lake Victoria, A = Lake Albert, E = Lake Edward, G = Lake George, EU = ellipsoidal uncertainty, OLU = optical level uncertainty, WLU = water level reading uncertainty.

The most significant uncertainty is likely the earth gravitational model conversion uncertainty (EGMu) introduced by using the tide-free spherical harmonic coefficients^16^, and this uncertainty remains unknown for discrete locations. The stated goal of EGM 2008 allows for ±15 cm global Root Mean Square geoid undulation commission error, and areas with quality gravitational data have been estimated as having uncertainties between ±5 and ±10 cm^16^. Lake Albert, Edward, and George have unknown EGMU uncertainties. The LEU for Lake Victoria is 2.5 cm, which aligns with the reported uncertainty of the Jason-3 altimeter^42^, and the altimeter data is already calibrated to EGM 2008^17^. EGMU is likely already incorporated into the LEU for Lake Victoria.

The LEU for Lake Albert is 0.05 m plus the additional unknown EGMU. The LEU for Lake Edward is 0.01 m plus the additional unknown EGMU. The LEU for Lake George is 0.16 m plus the additional unknown EGMU. The LEU for Lake Victoria is 2.5 cm.

### Lake sounding vertical uncertainty

The manufacturer provides the nominal accuracy of a 200 kHz transducer as: 0.01 m in addition to ±0.1 percent of depth. We use this value across all Lakes, although for a small portion of Lake Edward, the 33 kHz transducer was used, and Lake Victoria used a 120 kHz transducer. Table 5 gives the values of the echosounder uncertainty, Ues, for representative water depths. The maximum beam angle uncertainty, Uba, can be expressed in terms of depth using a simple trigonometric relation (Eq. 3), which results in the value in Table 5. A vessel’s pitch or roll will change the angle of the acoustic beam and introduce slant range errors in the measured depth. When the pitch or roll angle exceeds the transducer’s acoustic beam width, signal loss is likely to occur. When this occurred, the digitization process filled in the gaps. The acoustic beam width of the signal from the 200 kHz transducer is 5 deg. Vessel pitch rarely exceeded a few degrees, but roll angle could exceed 5 deg for short periods on unusually choppy days.

**Table 5:** Sounding Uncertainty.

Depth (m)	Ues (m)	Uba (m)	Utd (m)	Usv (m)	Usc (m)
1	0.01	0.00	0.20	0.00	0.20
10	0.02	0.04	0.20	0.04	0.21
20	0.03	0.08	0.20	0.08	0.23
30	0.04	0.11	0.20	0.12	0.26
40	0.05	0.15	0.20	0.17	0.31
50	0.06	0.19	0.20	0.21	0.35
60	0.07	0.23	0.20	0.25	0.40
70	0.08	0.27	0.20	0.29	0.45
80	0.09	0.30	0.20	0.33	0.50
90	0.10	0.34	0.20	0.37	0.55
100	0.11	0.38	0.20	0.42	0.61
110	0.12	0.42	0.20	0.46	0.66
115	0.13	0.44	0.20	0.48	0.69

Eq. 3 Beam Angle Uncertainty

$$\begin{array}{lll}{\rm{Uba}} & = & D\ast (1-(cos\;{5}^{\circ }))\\  &  & \,\therefore \\ {\rm{Uba}} & = & D\ast ({\rm{1}}-0.9962)\\  &  & \,\therefore \\ {\rm{Uba}} & = & D\ast 0.0038\end{array}$$

The transducer used on Lake Victoria uses a permanent fixed mount, so only a single measure of the initial draft below the water level (TD0) is required. Across all other Lakes, the TD0 was measured in calm water to within 1 cm each day when the mounting assembly was installed on the boat. On some occasions, TD0 had to be measured when the water surface was affected by small waves. Another impact on draft uncertainty was variations in the vessel’s draft with speed and, to some extent, by changes in displacement as fuel was used. Vessel rotational motions will also result in variance in draft. Attempts were made to keep redistribution of the equipment and crew movement to a minimum to minimize this effect, but this occasionally proved difficult in practice.

The upper limit of the TD variance is physically controlled by the boat construction and the transducer mounting method. It was observed that, when running at survey speed, any vessel motion which changed the draft by more than about + 20 cm would either raise the transducer out of the water, which would have been immediately apparent to the hydrographer, or caused water to overflow the transom, which would have been immediately evident to the crew. To allow for these uncertainties, the maximum transducer draft uncertainty, Utd, will is estimated at the physical limits of the range of transducer motion, or 20 cm.

Measured depth is a linear function of sound velocity in water. Sound velocity in freshwater is primarily affected by water temperature: lower temperatures result in slower velocities, and higher temperatures result in higher velocities. Standardized formulas provide accurate sound velocities as a function of temperature. Since no temperature profiles were collected during the surveys, all soundings were post-calibrated using the simplifying assumption of a single, constant sound velocity.

After the soundings were calibrated, various measurements of the actual temperatures from the four lakes surveyed were used to estimate the maximum abounding velocity uncertainty, Usv, due to variations in the sound velocity. The maximum measured temperatures spanned from 21.60 °C in Lake Victoria in 2018 to 30.1 °C in Lake Albert in 2020^44^. This range corresponds to a sound velocity ranging from 1486 m/sec to 1508 m/sec. Since the measured depth is linearly dependent on sound velocity, the Usv has the same percentages as the errors in the sound velocity, which are between −1.1 percent and 0.4 percent. The single value of Usv as a function of representative depths, reported in Table 5, is estimated as the maximum of the absolute range of ±Usv, or 1.1 percent.

Again, the combined sounding depth uncertainty (Usc) is calculated using the Root Sum of the Squares GUM methodology^43^ (Eq. 4). Unaccounted error relates to the decreasing fuel load during the day and other boat stability parameters such as the number and location of the crew members.

Eq. 4 - Sounding Uncertainty

$$Usc=\sqrt{Ue{s}^{2}+Ub{a}^{2}+Ut{d}^{2}+US{v}^{2}}$$

Across several days during the Lake Albert deployment, the GNSS L-band corrective signal became intermittent, dropping out for approximately 20 percent of the time. As a result, across Lake Albert, there were 59,287 GPS coordinate pairs without a differential signal and 233,504 GPS coordinate pairs with a differential signal. A decision was made to retain the 59,287 GPS coordinate pairs without a differential signal. The methodology below was designed to quantify this difference and guide the decision to include the uncorrected GPS data alongside the corrected GPS data. It should be noted that this drop of the L-band signal will only alter the horizontal position of soundings, as the depth is taken from hydroacoustic equipment, and the SDp was ascertained using a gauge and benchmark.

Two sample transects totaling 34 linear kilometers with an intermittent loss of differential correction were extracted from the entire data population. First, all 3,816 horizontal coordinate pairs on these two transects with a differential correction signal were connected linearly. This connection is defined as the line of best fit. It is merely a Euclidean connector between each pair of corrected GPS coordinate pairs. Next, the 4,714 GPS horizontal coordinate pairs without differential correction present were added to this line. These uncorrected points did not fit exactly on the best fit line but are often situated on either side of this line. Finally, the distance from the GPS horizontal coordinate pairs without differential corrections to the linear feature created from the GPS horizontal coordinate pairs with differential correction was calculated. The mean offset of the uncorrected GPS horizontal coordinate pairs from the corrected GPS horizontal coordinate pairs was 0.13 m. The maximum difference found across the 34 km sample is only 1.45 m. As no binning method should attempt to analyze these data at the sub-meter horizontal level, the uncorrected horizontal coordinate pairs were retained.

### Lake shorelines accuracy and uncertainty

We sample the shorelines using a higher resolution depiction of the shorelines; in this case, a UAS-derived shoreline. We operate under the assumption that the UAS operating at the 10 cm resolution locates the shoreline more accurately than a space-based system operating at 10 m or 30 m resolution. Using higher resolution data is a conventional method to obtain a map feature’s locational accuracy. The UAS was flown along 102 linear km of the Ugandan portion of the Lake Albert shoreline between February 1^st^ and February 20^th,^ 2021, and processed into a linear shoreline using the same method as the original lake shoreline.

All of the original vertices from the completed shoreline for Lake Albert were extracted. The vertices outside of the UAS-flight area were discarded. The original shoreline’s vertices’ locations were then compared against their distance to the UAS shorelines. Vertices are chosen as the anchors as these are the location an analyst determines that the shoreline is present by dropping a point; the shoreline itself is merely the mechanical connection of all the vertices placed by the analyst. The original shoreline’s vertices were within *14.46* *m* ± *0.52 (CI 95, n* = *8826)* of the UAS shorelines. That is, the average difference of the shorelines is well within one and a half Sentinel-2 pixels. This level of shoreline uncertainty applies to Lake Albert, Edward, and George. If Lake Victoria has the same level of shoreline uncertainty as the other three Lakes, this will equate to 21.69 m for Lake Victoria.

## Data Visualization

**Figure:**
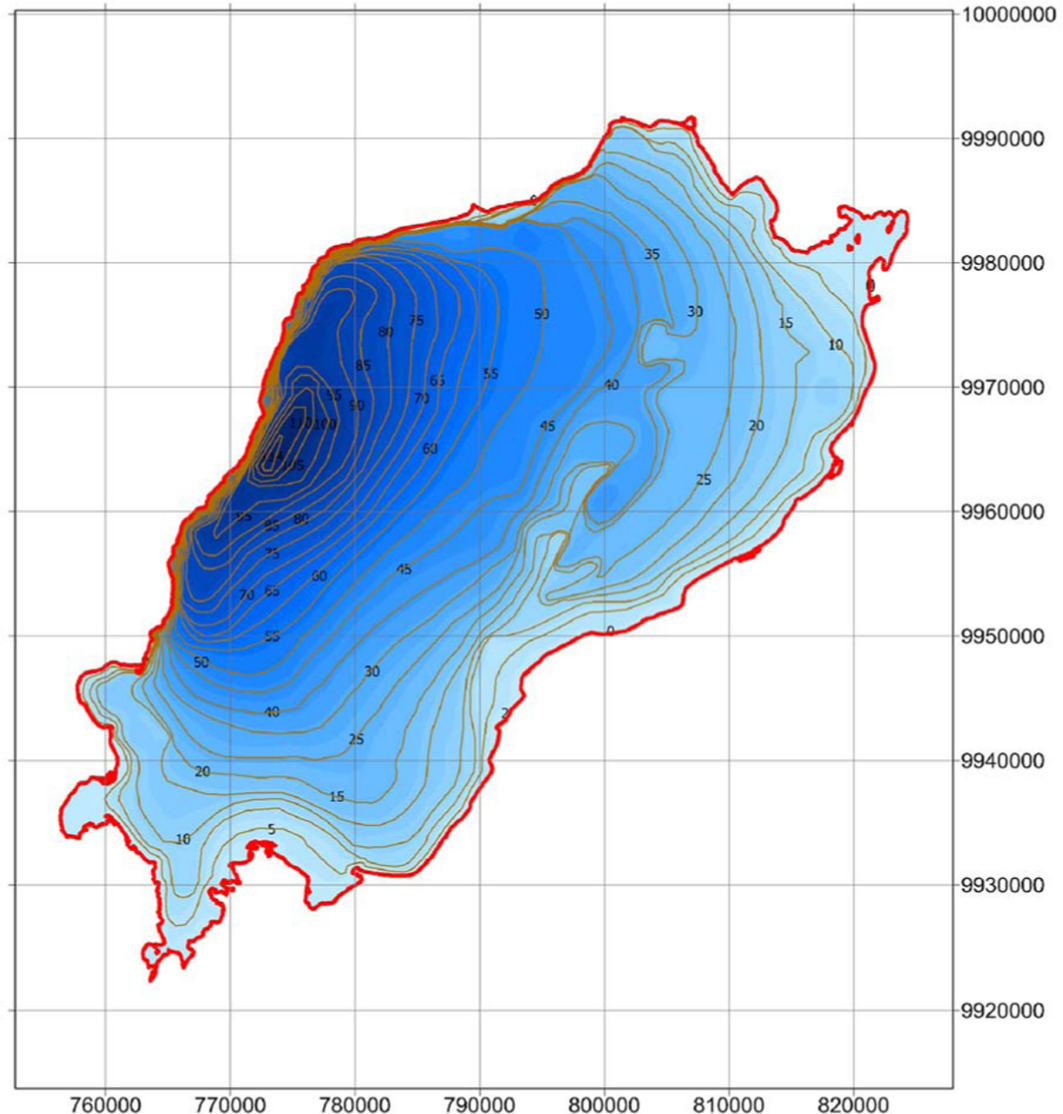
Lake Edward.

**Figure:**
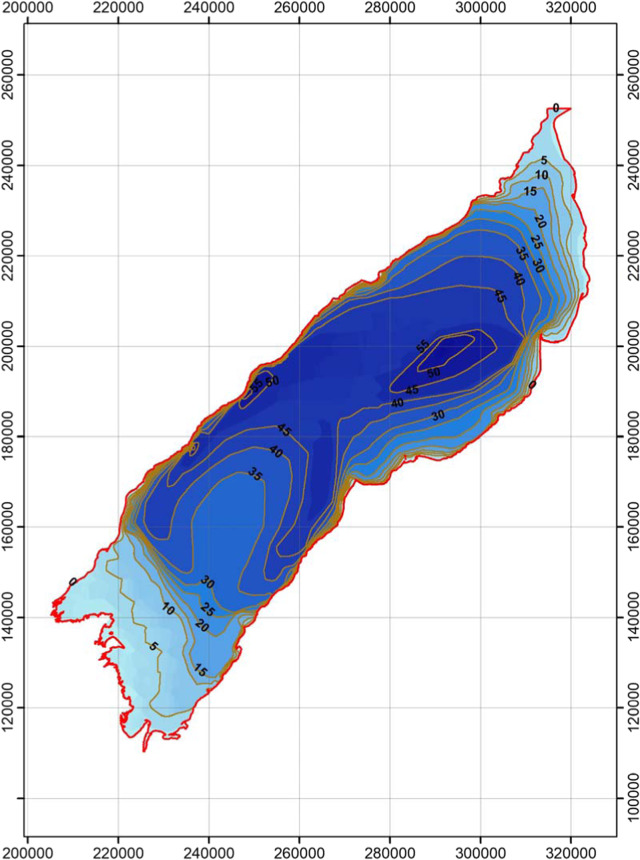
Lake Albert.

**Figure:**
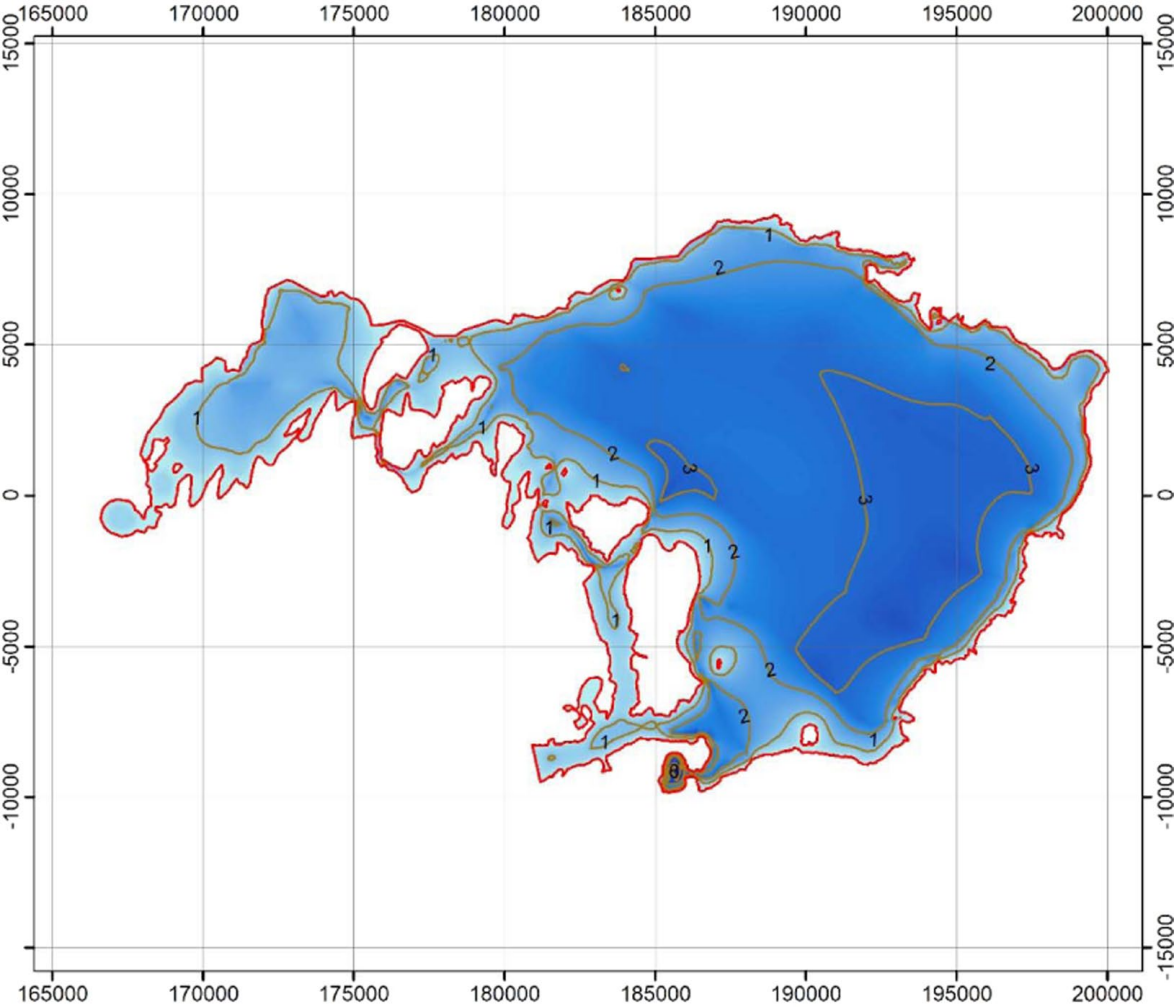
Lake George.

**Figure:**
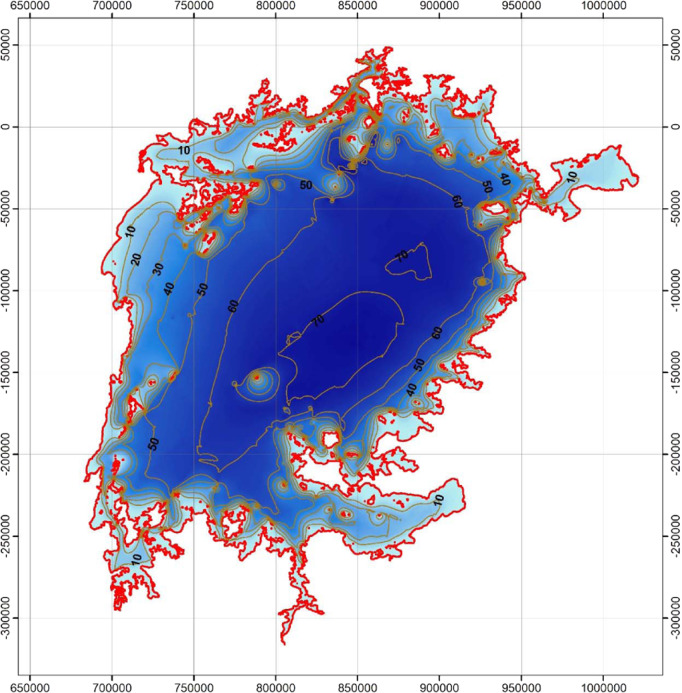
Lake Victoria.

## Usage Notes

All horizontal or vertical measurement units are meters, square meters, or cubed meters unless otherwise noted.

Unless otherwise noted, Lake Victoria’s geospatial data is referenced to EPSG:102024, and Lake Albert’s geospatial data is referenced to EPSG:32636. Lake Edward’s geospatial data is referenced to

EPSG:32735. Lake George is split between EPSG:32636 and EPSG:32736, but EPSG:32636 is used as the benchmark location, and most of the Lake is in the northern hemisphere. Therefore, certain software may read the coordinates for Lake George as out-of-bounds; in this scenario, convert Lake George to EPSG:102024 or another suitable coordinate system or use latitude and longitude as an alternate. Unless otherwise noted, vertical data is referenced to the official EGM 2008 WGS 1984 version^16^.

These data are packaged in open formats and ready for direct use in FOSS-GIS software such as QGIS, GRASS, GDAL/OGR, and commercial packages such as ArcGIS, GeoMedia, and Manifold. We encourage future researchers to continue to refine the Lake George benchmark and Lake Elevation Level provided.

## Data Availability

Fortran Code to generate the WGS 84 geoid undulations using spherical harmonic synthesis of EGM2008 is available from the NGA^15^ and is available for download at https://earthinfo.nga.mil/index.php?dir=wgs84&action=wgs84 and is linked from the repository. C++ Code to generate the WGS 84 geoid undulations of EGM 84 and EGM 96 is part of the GeographicLib project^45^ and is available for download https://geographiclib.sourceforge.io/html/GeoidEval.1.html. The Java code to repeat the volume and mean depth calculations is deposited in the GitHub Tinfour repository located at https://github.com/gwlucastrig/Tinfour and is linked from the repository.

## References

[1] Chorowicz J (2005). The east African rift system. Journal of African Earth Sciences.

[2] Bastawesy ME, Gabr S, White K (2013). Hydrology and geomorphology of the Upper White Nile Lakes and their relevance for water resources management in the Nile basin. Hydrological Processes.

[3] Lawson FH (2017). Egypt versus Ethiopia: the conflict over the Nile Metastasizes. The International Spectator.

[4] Nakiyende, H. *et al*. Regional catch assessment survey of 2019 for Lakes Edward and Albert (D.R Congo and Uganda). 1–38 (Nile Equatorial Lakes Subsidiary Action Program (NELSAP-CU)/Nile Basin Initiative (NBI), Kigali, Rwands, 2019).

[5] von Sarnowski, A. In Proceedings: Conference on International Agricultural Research for Development. www.tropentag.de/de/2004/proceedings/node402.html.

[6] Hamilton SE (2020). The use of unmanned aircraft systems and high-resolution satellite imagery to monitor tilapia fish-cage aquaculture expansion in Lake Victoria, Kenya. Bulletin of Marine Science.

[7] Njiru J, van der Knaap M, Kundu R, Nyamweya C (2018). Lake Victoria fisheries: Outlook and management. Lakes & Reservoirs: Research & Management.

[8] Aura CM (2020). Checking the pulse of the major commercial fisheries of lake Victoria Kenya, for sustainable management. Fisheries Management and Ecology.

[9] Balirwa, J. S. *et al*. Biodiversity and Fishery Sustainability in the Lake Victoria Basin: An Unexpected Marriage? *BioScience* **53**, 703–715, 10.1641/00063568(2003)053[0703:Bafsit]2.0.Co;2 (2003).

[10] Sayer, C. A., Máiz-Tomé, L. & Darwall, W. *Freshwater biodiversity in the Lake Victoria Basin:Guidance for species conservation, site protection, climate resilience and sustainable livelihoods*. (International Union for Conservation of Nature, 2018).

[11] Kaufman L (1992). Catastrophic change in species-rich freshwater ecosystems. BioScience.

[12] Goldschmidt T, Witte F, Wanink J (1993). Cascading effects of the introduced Nile perch on the detritivorous/phytoplanktivorous species in the sublittoral areas of Lake Victoria. Conservation biology.

[13] Marshall BE (2018). Guilty as charged: Nile perch was the cause of the haplochromine decline in Lake Victoria. Canadian Journal of Fisheries and Aquatic Sciences.

[14] Witte F (1992). The destruction of an endemic species flock: quantitative data on the decline of the haplochromine cichlids of Lake Victoria. Environmental biology of fishes.

[15] Harmonic Synth WGS84 -A FORTRAN program to compute geoid heights with respect to WGS 84 by spherical harmonic synthesis. v. 6032008 (NGA, Washington DC, 2008).

[16] Pavlis, N. K., Holmes, S. A., Kenyon, S. C. & Factor, J. K. The development and evaluation of the Earth Gravitational Model 2008 (EGM2008). *Journal of geophysical research: solid earth* **117** (2012).

[17] Birkett CM (2018). G-REALM: A Lake/Reservoir Monitoring tool for Water Resources and Regional Security assessment. AGUFM.

[18] NASA: Jet Propulsion Laboratory. *U.S. Releases Enhanced Shuttle Land Elevation Data*, http://www2.jpl.nasa.gov/srtm/ (2016).

[19] Picot, N. *et al*. Vol. 1 (eds CNES *et al*.) 71 (CNES, Toulouse, France, 2018).

[20] LVFO. Revised Standard Operating Procedures for Hydro-acoustics surveys on Lake Victoria. (Lake Victoria Fisheries Organization, Jinja, Uganda, 2018).

[21] Echoview. Line picking algorithms: Best bottom candidate, https://support.echoview.com/WebHelp/Reference/Algorithms/Line_picking_algorithm.htm#Best_bottom_candidate (2021).

[22] International Lake Environment Committee. World lakes database. *As viewed online at* http://www.ilec. *or. jp/database/database. html* (2001).

[23] European Space Agency. *Copernicus Open Access Hub*, https://sentinels.copernicus.eu/web/sentinel/missions/sentinel-2 (2019).

[24] European Space Agency. *Sentinel-2: Land Overview*, https://sentinel.esa.int/web/sentinel/thematic-areas/land-monitoring (2019).

[25] Drusch M (2012). Sentinel-2: ESA’s optical high-resolution mission for GMES operational services. Remote sensing of Environment.

[26] Tobler, W. in *Proceedings of the International Workshop on Geographic Information Systems*. (International Geographic Union, Commission on Geographical Information…).

[27] Wessel, P. & Smith, W. GSHHG—A Global Self-Consistent, Hierarchical, High-Resolution Geography Database. *Honolulu, Hawaii, Silver Spring, Maryland*. (*URL**:* http://www.soest.hawaii. edu/pwessel/gshhg*/**(accessed 10 January2013)* (2013).

[28] ArcGIS Pro 2.7 (ESRI, Redlands, CA, 2021).

[29] Geospatial Data Abstraction Library (Open Source Geospatial Foundation, Dover, DE, 2016).

[30] QGIS (Open Source Geospatial Foundation, Dover, DE, 2017).

[31] eBee Plus Drone User Manual Revision 1.7 (senseFly, Cheseaux-Lausanne, Switzerland, 2018).

[32] senseFly S.O.D.A. Camera User Manual Revision 1.5 (senseFly, Cheseaux-Lausanne, Switzerland, 2017).

[33] eMotion 3 User Manual, Revision 1.9 (senseFly, Cheseaux-Lausanne, Switzerland, 2018).

[34] Pix4D SA. 305 (Pix4D SA, Lausanne, Switzerland, 2017).

[35] Tinfour- High-Performance 2D Delaunay Triangulation and Related Utilities Written in Java (San Francisco, CA, 2021).

[36] Hamilton SE (2021). Harvard Dataverse.

[37] Hamilton SE (2021). Harvard Dataverse.

[38] Hamilton SE (2021). Harvard Dataverse.

[39] Guyer, P. An Introduction to Accuracy Standards for Land Surveys. 1–35 (2017).

[40] Cretaux J-F (2018). Absolute calibration or validation of the altimeters on the Sentinel-3A and the Jason-3 over Lake Issykkul (Kyrgyzstan). Remote Sensing.

[41] Biancamaria S (2018). Validation of Jason-3 tracking modes over French rivers. Remote Sensing of Environment.

[42] NASA Jet Propulsion Laboratory. *Jason-3: Summary*, https://sealevel.jpl.nasa.gov/missions/jason-3/summary/ (2021).

[43] Bipm, I., Ifcc, I., Iso, I. & Iupap, O. Evaluation of measurement data—guide to the expression of uncertainty in measurement, JCGM 100: 2008 GUM 1995 with minor corrections. *Joint Committee for Guides in Metrology* (2008).

[44] Lubbers J, Graaff R (1998). A simple and accurate formula for the sound velocity in water. Ultrasound in medicine & biology.

[45] GeographicLib: Evaluate the Geoid Height (2020).

[46] Miriti, E. A. K. Lake Albert. *African Great Lakes Information Platform*. https://www.africangreatlakesinform.org/page/lake-albert (2017).

[47] Seders LA, Shea CA, Lemmon MD, Maurice PA, Talley JW (2007). LakeNet: an integrated sensor network for environmental sensing in lakes. Environmental Engineering Science.

[48] Tong X (2016). Estimating water volume variations in Lake Victoria over the past 22 years using multi-mission altimetry and remotely sensed images. Remote Sensing of Environment.

